# N6-methyladenosine modification of NEU1 mediated by METTL3 exacerbates angiotensin II-induced atrial fibrillation

**DOI:** 10.1080/19336918.2026.2650272

**Published:** 2026-03-31

**Authors:** Zhilan Zhang, Yanbing Luo, Qiuguo Zou, Zelai Mo

**Affiliations:** Department of Ultrasonic Medicine, Haikou Affiliated Hospital of Central South University Xiangya School of Medicine, Haikou, P. R. China

**Keywords:** Atrial fibrillation, atrial fibrosis, m6A modification, METTL3, NEU1

## Abstract

Neuraminidase 1 (NEU1) regulation of atrial fibrillation (AF) progression via fibrosis remains unknown. Mice receiving AAV9-mediated NEU1 knockdown were infused with Ang II and subjected to programmed electrical stimulation to induce AF. Left atrial dilation and fibrosis were evaluated by echocardiography, histology, and fibrosis markers. Primary mouse atrial fibroblasts treated with Ang II were assessed for proliferation and migration by EdU staining and Transwell. The N6-methyladenosine (m6A) modification of NEU1 by methyltransferase-like 3 (METTL3) was confirmed through m6A quantification, RNA immunoprecipitation, MeRIP-qPCR and actinomycin D experiments. NEU1 knockdown attenuated atrial dilation, fibrosis, and AF susceptibility. Mechanistically, METTL3 stabilized NEU1 via m6A modification, promoting Ang II‑induced atrial fibroblast activation. Thus, NEU1, stabilized by METTL3 via m6A, exacerbates Ang II‑induced AF susceptibility.

## HIGHLIGHTS

1. Ang II upregulates NEU1 expression in atrial tissues.
2. Knocking down NEU1 can inhibit the activation of atrial fibroblasts and thus alleviate Ang II-induced AF susceptibility and atrial fibrosis.
3. The expression of NEU1 is regulated by m6A modification mediated by METTL3.

## Introduction

Atrial fibrillation (AF), is one of the most prevalent cardiac arrhythmias, significantly impairing patients’ quality of life and predisposing them to severe complications such as stroke and heart failure [1]. The incidence of AF increases markedly after the age of 40, and with the global aging population, AF has become a substantial public health burden [2]. Current clinical management primarily relies on antiarrhythmic drugs and catheter ablation to alleviate AF and restore normal rhythm [3]. However, a significant proportion of patients experience recurrence and adverse outcomes, underscoring the urgent need to elucidate the underlying mechanisms of AF and identify novel therapeutic targets.

Atrial fibrosis, a hallmark of structural remodeling in the atria, is a key driver in AF pathogenesis [4]. It disrupts electrical conduction, slows conduction velocity, and facilitates reentry circuits, thereby promoting the initiation and perpetuation of AF [4]. Neuraminidase (NEU), also known as sialidase, is a family of enzymes that cleave sialic acids from cell surface glycoproteins and glycolipids [5]. In mammals, four primary isoenzymes (NEU1-4) exist, which act as critical ‘molecular switches’ by regulating the desialylation of glycoconjugates, thereby influencing essential physiological processes such as cell adhesion, signal transduction, and immune modulation [6]. Emerging evidence suggests that NEU enzymes, particularly neuraminidase 1 (NEU1), are implicated in cardiovascular and fibrosis related diseases [7]. For instance, elevated NEU1 expression occurs in chronic kidney disease patients and serves as a key mediator of renal fibrosis [8]. Similarly, in respiratory diseases, NEU1 exacerbates pulmonary fibrosis [9]. In the context of cardiovascular disease, NEU1 expression is upregulated in the hearts of mice following myocardial infarction, and its genetic ablation in cardiomyocytes attenuates myocardial remodeling, oxidative stress, and mitochondrial dysfunction [10]. Furthermore, pharmacological inhibition of NEU1 mitigates fibrosis, inflammation, apoptosis, and oxidative stress in diabetic cardiomyopathy by activating AMPKα and SIRT3 signaling pathways [10]. Despite these advances, the role of NEU1 in AF remains unexplored. Given its involvement in fibrosis across various tissues, we hypothesize that NEU1 May promote atrial fibrosis and contribute to AF pathogenesis.

Recent studies have highlighted the role of N6-methyladenosine (m6A) RNA modification, a dynamic and reversible epigenetic mechanism, in regulating gene expression and cellular processes [11]. This modification has also been reported to play a significant role in AF [12]. Notably, methyltransferase-like 3 (METTL3), a core component of the m6A methyltransferase complex, is upregulated in the hearts of AF patients [13]. METTL3-mediated m6A modification of Insulin-like Growth Factor Binding Protein 3 (IGFBP3) has been shown to drive cardiac fibroblast activation and fibrosis [14]. However, whether NEU1 expression is regulated by m6A modification remains unknown. Bioinformatic screening via SRAMP database (http://www.cuilab.cn/sramp) revealed stringent m6A modification targets in NEU1 mRNA, suggesting a potential regulatory role of m6A in NEU1 expression.

In light of this evidence, we present a hypothesis: NEU1 expression is regulated by METTL3-mediated m6A modification, and this regulatory axis exacerbates angiotensin II (Ang II)-induced AF susceptibility.

## Materials and methods

### Animals

All animal experiments were performed following approval from the Haikou Affiliated Hospital of Central South University Xiangya School of Medicine Animal Ethics Committee and complied with NIH standards for laboratory animal welfare (approval no. 2021–367). Eight-week-old male C57BL/6 mice were utilized in this investigation. Mice were randomly assigned to experimental groups using a random number table method, and the researchers responsible for subsequent animal handling, electrophysiological detection, histological staining and data analysis were blinded to the group allocation. Ang II (HY-13948, Medchemexpress, Shanghai, China) was delivered subcutaneously for 21 days at 2000 ng/kg/min using osmotic minipumps (Alzet model 1004; Durect, CA, USA) [15], while control animals received saline via identical pumps. After 3 weeks, subjects were humanely sacrificed via cervical dislocation under isoflurane-induced anesthesia, and atrial tissues were collected for further analysis. To achieve fibroblast-specific knockdown of NEU1, a recombinant AAV9 vector driven by the periostin (Postn) promoter was constructed as previously described [16]. The vector, designated AAV9-Postn-sh-NEU1, carries a short hairpin RNA (shRNA) targeting the mouse NEU1 gene under the transcriptional control of the mouse Postn promoter, which confers specific expression in cardiac fibroblasts. A control vector expressing a non-targeting shRNA (AAV9-Postn-sh-NC) was generated using the same backbone. Mice were injected with AAV9-Postn-sh-NC and AAV9-Postn-sh-NEU1 (3 × 10^8^ TU/mouse [17]) through tail vein 2 weeks before Ang II induction to achieve NEU1 knockdown.

### AF induction

On the 21st day of Ang II administration, animals were anesthetized by intraperitoneal delivery of 2.5% tribromoethanol (0.02 mL/g, T48402, MERCK, Germany). An octapolar electrode catheter (Japan Lifeline, Tokyo, Japan) was inserted for intracardiac electrophysiological evaluation. AF susceptibility was assessed by inducing AF via programmed electrical stimulation using a computer-controlled stimulator. Specifically, repetitive burst pacing trains of 5-second duration were delivered through the distal electrodes. The initial pacing cycle length was set at 40 ms and progressively shortened by 2-ms decrements in subsequent bursts until a minimum cycle length of 20 ms was achieved, consistent with established arrhythmia induction paradigms [18]. AF was operationally defined as an irregular atrial rhythm with a minimal duration of ≥1 second, characterized by the absence of distinct P waves on the electrogram, irregular RR intervals, and coarse or fragmented atrial electrograms. Atrial tachycardia (AT) and atrial flutter (AFL) were distinguished from AF by their regular rhythm: AT/AFL exhibited consistent cycle lengths and uniform atrial waveforms (sawtooth waves for AFL, monomorphic waves for AT), whereas AF showed completely disorganized atrial activity without predictable rhythm.

### Echocardiography

Echocardiographic analysis of left atrial diameter was carried out on the VisualSonics Vevo 2100 platform (Toronto, Canada). Mice were anesthetized with isoflurane, and M-mode images were obtained to measure left atrial dimensions.

### Quantitative reverse transcription polymerase chain reaction (qPCR)

Atrial tissue and cellular RNA were isolated with a commercial extraction kit (RC113-01, Vazyme, Nanjing, China). RNA concentration and purity were determined using a NanoDrop spectrophotometer (Thermo Fisher Scientific, Waltham, MA, USA). Only samples with an A260/A280 ratio between 1.8 and 2.1 and an A260/A230 ratio greater than 2.0 were used for subsequent experiments. Reverse transcription was carried out with a cDNA Synthesis Kit (MT404, Biomed, Shanghai, China). qPCR assays were conducted using SYBR Green reagents (MT561, Biomed) on a ABI7900-HT-Fast system (Applied Biosystems, Foster, USA). Primer sequences are listed in Table 1, and transcript levels were normalized using the 2^−ΔΔCt^ method.

**Table 1.** Primers employed in this study.

Genes	5’–>3’
NEU1 (Mouse) F	CTGTCTCCTCAGTGATGACCAC
NEU1 (Mouse) R	ATGACCGAGCCATCTGGAAGCT
α-SMA (Mouse) F	CGTGGCTATTCCTTCGTGACTACTG
α-SMA (Mouse) R	CGTCAGGCAGTTCGTAGCTCTTC
Collagen I (Mouse) F	CCTCAGGGTATTGCTGGACAAC
Collagen I (Mouse) R	CAGAAGGACCTTGTTTGCCAGG
GAPDH (Mouse) F	CATCACTGCCACCCAGAAGACTG
GAPDH (Mouse) R	ATGCCAGTGAGCTTCCCGTTCAG

### Methylated RNA immunoprecipitation (MeRIP)-qPCR

Total RNA was extracted using a commercial extraction kit (RC113-01, Vazyme) and fragmented into 200–300 nt fragments with RNA fragmentation buffer (AM8740, Thermo Fisher Scientific). For each sample, 50 μg of fragmented RNA was reserved as input, and the remaining RNA was incubated with 5 μg of anti-m6A antibody (68055–1-Ig, Proteintech) or IgG antibody (30000–0-AP, Proteintech) at 4°C overnight with gentle rotation, followed by incubation with Protein A/G magnetic beads (88802, Thermo Fisher Scientific) for 2 hours at 4°C. The beads were washed sequentially with low-salt and high-salt buffers, and the immunoprecipitated RNA was eluted and purified using an RNA purification kit (ER701, Transgene, China). The purified RNA (IP and input samples) was reverse-transcribed into cDNA with a cDNA Synthesis Kit (MT404, Biomed), and qPCR was conducted using SYBR Green reagents (MT501, Biomed) on an ABI7900-HT-Fast system (Applied Biosystems, Foster, USA) with NEU1 primers. The relative m6A modification level of NEU1 mRNA was calculated as the ratio of IP to input, normalized to the Control group.

### Western blot

Atrial tissues/cells were homogenized/lysed in RIPA buffer. Protein concentrations were determined using the BCA assay (E-BC-K318-M, Elabscience, Wuhan, China). Equal amounts of protein (30 µg) were separated by SDS-PAGE and transferred to PVDF membranes (F619534, Sangon, Shanghai, China). Membranes were blocked with 5% nonfat milk and incubated overnight at 4°C with primary antibodies. After incubation with HRP-conjugated secondary antibodies, protein signals were detected with an ECL substrate (PH0353, PHYGENE, Fuzhou, China) and band intensities were measured with ImageJ software. Protein bands were normalized relative to GAPDH as a reference. Western blot was performed using the following primary antibodies: anti-NEU1 (Proteintech, 67,032–1-Ig, 1/1000), anti-α-SMA (Affinity, AF1032, 1/1000), anti-Collagen I (Affinity, AF7001, 1/1000), anti-METTL3 (Affinity, DF12020, 1/1000), and anti-GAPDH (Abcam, ab8245, 1/10000) as loading control, followed by incubation with corresponding secondary antibodies (goat anti-rabbit, Abcam, ab205718; goat anti-mouse, Abcam ab205719, 1/10000).

### Immunofluorescence (IF)

Atrial tissues underwent fixation with 4% paraformaldehyde (PH0427, PHYGENE), followed by paraffin embedding and microtome sectioning (5 µm). After xylene-mediated paraffin removal and graded alcohol rehydration, heat-induced epitope retrieval was performed. Nonspecific binding was blocked with 5% BSA before overnight exposure to primary antibody targeting NEU1 (67032–1-Ig, Proteintech) overnight at 4°C. The next day, sections were washed and incubated with a fluorescently labeled secondary antibody for 1 hour at room temperature in the dark. Nuclei were counterstained with DAPI (PH0524, PHYGENE) for 5 minutes. Finally, slides were mounted with antifade mounting medium (P0128, Beyotime) and sealed. Images were captured using a fluorescence microscope (Olympus IX83).

### Histological analysis

Atrial fibrosis was assessed using Masson staining (BL1059A, Biosharp, Hefei, Anhui) and Picrosirius red staining (BL1194A, Biosharp). Atrial specimens were preserved in 4% PFA, processed for paraffin embedding, and cut into 5-μm sections. Following standard staining protocols, the fibrotic area (highlighted in blue by Masson staining and in red by Picrosirius red staining) was quantified in a blinded manner using ImageJ software.

### Primary atrial fibroblast isolation and transfection

Atrial fibroblasts were isolated from mouse by enzymatic digestion and cultured in DMEM (11965092, Gibco, MA, USA) supplemented with 10% FBS (A5256701, Gibco) as previously described [19]. The short hairpin RNAs (shRNAs) specifically targeting METTL3 or NEU1 were chemically synthesized by VectorBuilder (Guangzhou, China). The corresponding empty shRNA vectors were used as negative controls (sh-NC). For NEU1 overexpression (oe-NEU1) construction, the full-length NEU1 cDNA was cloned into the pcDNA3.1(+) expression vector. For transfection experiments, cells were plated at a density of 5 × 10^5^ cells per well in 6-well plates 24 hours prior to transfection to ensure optimal cell confluence. Using Lipofectamine-based transfection (L3000150, Thermo Fisher Scientific, Massachusetts, USA), we introduced sh-RNAs into primary atrial fibroblasts, which were then subjected to 24 h Ang II treatment (1 μM) [20].

### M6A level detection

RNA samples from atrial tissues and fibroblasts were prepared for m6A level measurement with a commercial methylation assay kit (ab185912, abcam), following standard protocols.

### 5-ethynyl-2′-deoxyuridine (EdU) assay

The proliferation of cells was assessed using the Click-iT EdU Imaging Kit (C0079, Beyotime, Shanghai, China). Cells were plated in 24-well plates (5,000 cells/well) and pulsed with EdU for 2 h. Following fixation (4% PFA) and membrane permeabilization (0.3% Triton X-100, ST797, Beyotime), samples underwent Click chemistry staining. Nuclei were counterstained with DAPI. The total number of cells (DAPI-positive nuclei) and the number of proliferating cells (EdU-positive cells) were counted using Nikon fluorescence microscopy. The proliferation rate for each field was calculated as follows:

(Number of EdU-positive nuclei/Total number of DAPI-positive nuclei) × 100%. The average percentage from the five fields of view was then taken as the final value for that independent experimental replicate.

### Transwell assay

The migration of cells was evaluated in Corning Transwell systems (8 μm pores). Cells (1 × 10^5^) suspended in serum-deprived medium were placed in the upper compartment, while the lower compartment held complete medium. Following 24-hour incubation, membranes with traversed cells were methanol-fixed, labeled with 0.1% crystal violet (G1062, Solarbio, Beijing, China), and quantified microscopically.

### RNA immunoprecipitation (RIP)

RIP assays were conducted with a RIP kit (FI8703, Fitgene, Guangzhou, China). Cellular lysates from 2 × 10^7^ cells underwent immunoprecipitation with METTL3-specific antibody (Abcam, ab195332), followed by qPCR analysis of co-precipitated NEU1 transcripts.

### Actinomycin D assay

NEU1 transcript stability was evaluated by actinomycin D assay (5 μg/mL, M4849, Abmole, Shanghai, China) for 0, 3, and 6 hours. Total RNA was extracted, and NEU1 mRNA levels were quantified by qPCR. mRNA half-life was calculated using linear regression analysis.

### Statistical analysis

Data processing utilized GraphPad Prism 10.1.2, with continuous variables expressed as mean ± SD. Intergroup differences were assessed via two-tailed t-tests, while multigroup comparisons employed one-way ANOVA with Tukey’s correction. Statistical significance was defined as *p* < .05. Detailed statistical results, including group comparisons, test statistics, and P values for the figures presented in the main text, are provided in Supplementary Table S1–S22.

## Results

### Ang II infusion upregulated NEU1 expression in mouse atria

**Figure 1.**
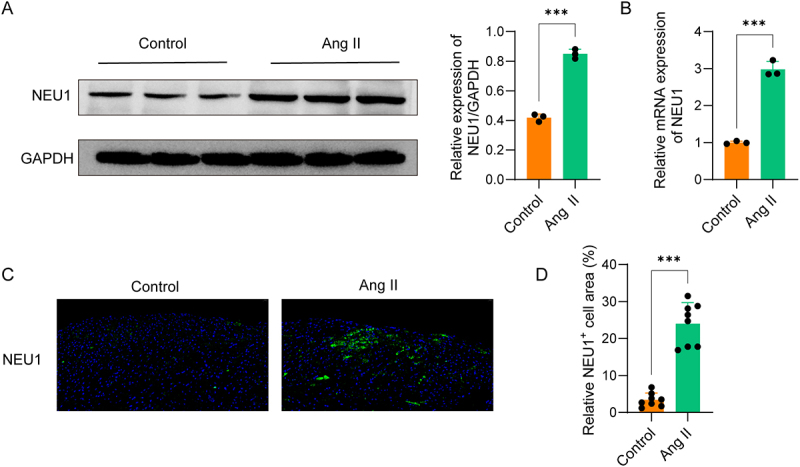
Ang II infusion upregulated NEU1 expression in mouse atria. (A-B) The expression of NEU1 in atrial tissues of mice (*n* = 8) was detected by western blot and qPCR. (C-D) Immunofluorescence analysis was performed to detect and quantify NEU1 expression in mouse atrial tissues.

Atrial NEU1 levels were markedly elevated in Ang II-infused mice compared with saline-infused controls (Figure 1A–B). IF analysis further corroborated these findings (Figure 1C–D), implicating NEU1 in Ang II-induced AF susceptibility development through its enhanced expression during Ang II stimulation.

### NEU1 knockdown attenuated Ang II-induced AF and atrial fibrosis

**Figure 2.**
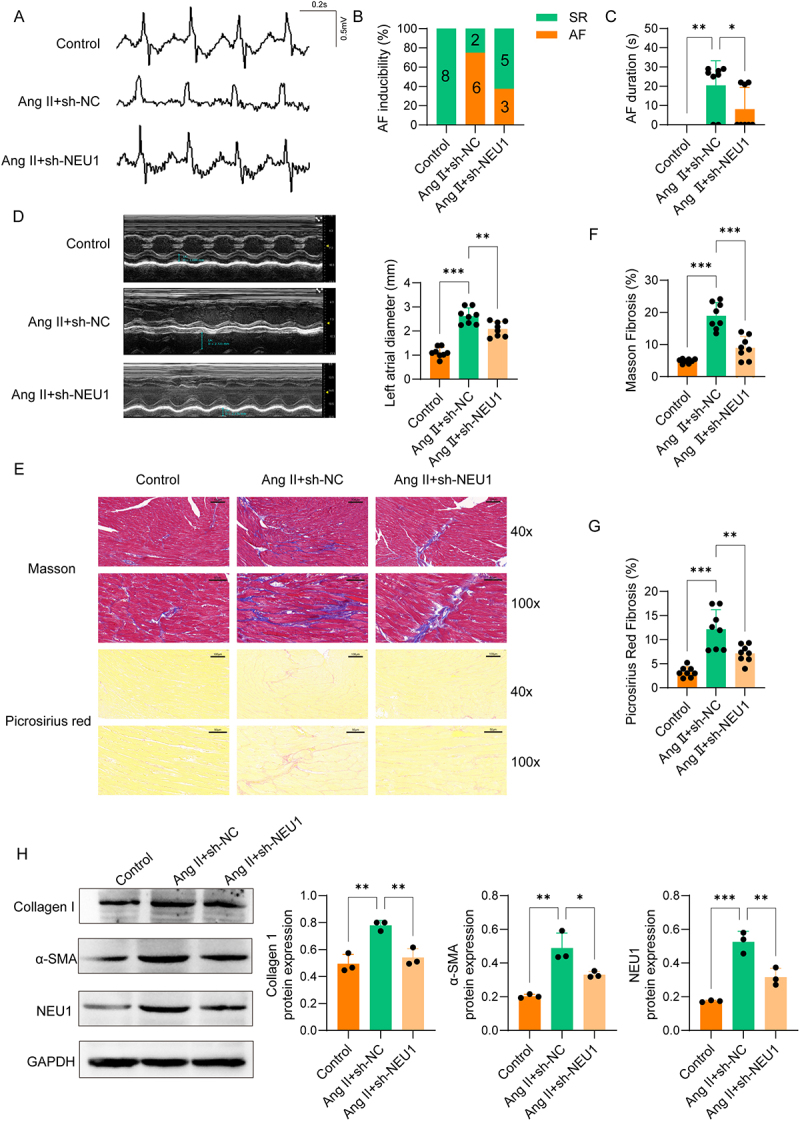
NEU1 knockdown attenuated angiotensin II-induced AF and atrial fibrosis mice (*n* = 8) administered either AAV9-sh-NC and AAV9-periostin promoter-sh-NEU1 via tail vein injection were subjected to Ang II infusion. (A) Representative atrial electrograms on day 21. (B) Percentage of mice exhibiting successful AF induction in each group. (C) AF duration in each group. (D) Echocardiographic assessment of left atrial dilation on day 21. (E-G) Representative images of Masson staining and Picrosirius red staining in atrial sections, and quantitative analysis of fibrotic areas. (H) Western blot analysis of NEU1 and fibrosis markers (α-SMA and collagen I) in atrial tissues on day 21.

To elucidate NEU1’s involvement in AF susceptibility, mice were first administered AAV9-sh-NC and AAV9-sh-NEU1 via tail vein injection. Two weeks after viral delivery, Ang II was continuously infused subcutaneously via osmotic minipumps to promote susceptibility to AF. AF susceptibility was then assessed by in vivo electrophysiological study with programmed electrical stimulation. In the sh-NC+Ang II group (*n* = 8), AF was successfully induced in 6 mice (75%), a marked increase compared to the saline-treated controls (0 out of 8, 0%) (Figure 2A–B). However, NEU1 knockdown (sh-NEU1+Ang II group, *n* = 8) reduced the AF inducibility to 3 mice (37.5%) (Figure 2A–B). Furthermore, the AF duration was shorter in Ang II+sh-NEU1 group relative to Ang II+sh-NC group (Figure 2C).

Cardiac structural alterations, a well-established contributor to AF susceptibility, were evaluated following NEU1 suppression. Echocardiographic analysis demonstrated pronounced left atrial dilation post-Ang II challenge, while NEU1 knockdown attenuated Ang II-induced left atrial dilation (Figure 2D). Atrial fibrosis, a hallmark of atrial remodeling, was significantly increased after Ang II induction as shown by Masson staining and Picrosirius red staining, but NEU1 knockdown effectively reduced fibrotic areas (Figure 2E–G). At the protein level, NEU1 and fibrosis markers (Collagen I and α-SMA) were significantly elevated in the Ang II+sh-NC group compared to controls, while sh-NEU1 effectively suppressed NEU1 expression and reduced fibrosis marker levels (Figure 2H). These findings demonstrate that NEU1 knockdown inhibits Ang II-induced AF susceptibility, left atrial dilation, and fibrosis.

### NEU1 knockdown suppressed Ang II-induced activation of atrial fibroblast

**Figure 3.**
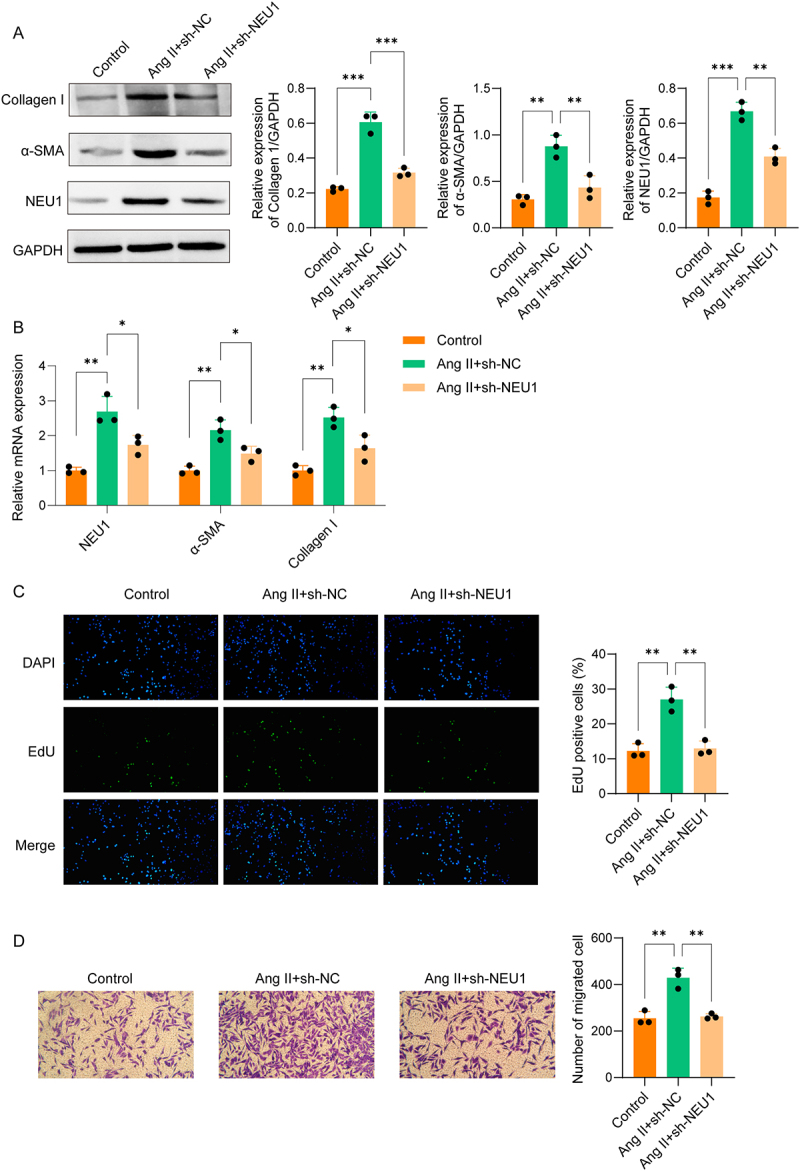
NEU1 knockdown suppressed Ang II-induced activation of atrial fibroblast. (A-B) Western blot and qPCR analyses were performed to detect NEU1, α-SMA, and collagen I expression in mouse atrial fibroblasts. (C) EdU staining was used to assess proliferation of atrial fibroblast. (D) Transwell assay was used to evaluate migration of atrial fibroblast.

To simulate AF *in vitro*, mouse atrial fibroblasts were treated with Ang II. Ang II induction significantly increased NEU1 and fibrosis marker expression in atrial fibroblasts, which was reversed by sh-NEU1 (Figure 3A–B). Additionally, Ang II treatment increased the number of EdU-positive cells and enhanced cell migration, both of which were reduced by NEU1 knockdown (Figure 3C–D). Experimental evidence reveals that inhibition of NEU1 suppresses Ang II-induced activation of atrial fibroblasts.

### NEU1 mRNA stability is regulated by m6A modification

**Figure 4.**
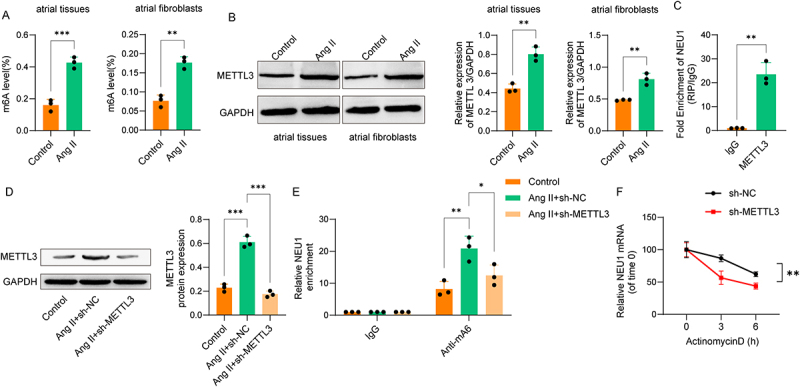
NEU1 mRNA stability is regulated by m6A modification. (A) Total m6A levels in RNA from mouse atrial tissues and atrial fibroblasts were measured using an m6A quantification kit. (B) Western blot analysis of METTL3 expression in atrial tissues and atrial fibroblasts. (C) RNA immunoprecipitation (RIP) assay confirming the interaction between NEU1 and METTL3 in atrial fibroblasts. (D) Western blot analysis of METTL3 expression in primary mouse atrial fibroblasts. (E) MeRIP-qPCR assay detecting the m6A modification level on NEU1 mRNA in primary mouse atrial fibroblasts. (F) NEU1 mRNA stability assessed by qPCR in atrial fibroblasts treated with actinomycin D at 0, 3, and 6 hours post-transfection with sh-NC or sh-METTL3.

To investigate whether NEU1 expression in Ang II-induced AF susceptibility is regulated by m6A modification, we first measured m6A levels. We observed that Ang II induction increased total m6A levels in RNA from mouse atrial tissues and fibroblasts (Figure 4A), as well as upregulated METTL3 expression (Figure 4B). RIP assays confirmed the interaction between NEU1 and METTL3 (Figure 4C). Western blot results showed that sh-METTL3 effectively inhibited METTL3 expression in Ang II-stimulated atrial fibroblasts compared with the Ang II+sh-NC group (Figure 4D). Meanwhile, MeRIP-qPCR assays demonstrated that Ang II stimulation significantly elevated the m6A modification level on NEU1 mRNA, while this enrichment was markedly reversed by METTL3 knockdown (Figure 4E). Moreover, sh-METTL3 reduced the stability of NEU1 mRNA in atrial fibroblasts compared to sh-NC (Figure 4F). These findings suggest that NEU1 mRNA stability is regulated by m6A modification.

### METTL3 promoted Ang II-induced activation of atrial fibroblast via NEU1

**Figure 5.**
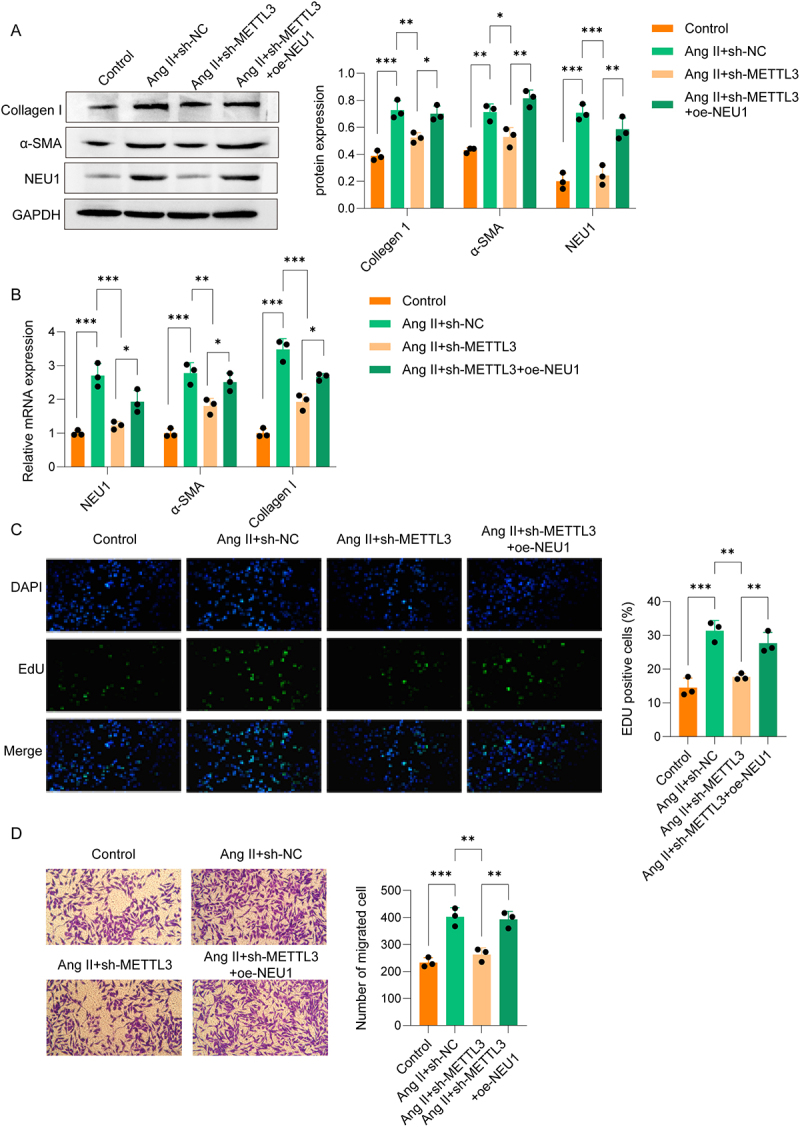
METTL3 promoted Ang II-induced activation of atrial fibroblast via NEU1 atrial fibroblasts were treated with Ang II and transfected with sh-METTL3 and/or oe-NEU1. (A-B) Western blot and qPCR analyses were performed to NEU1, α-SMA, and collagen I levels in mouse atrial fibroblasts. (C) EdU staining was used to assess proliferation of atrial fibroblast. (D) Transwell assay was used to evaluate migration of atrial fibroblast.

Finally, to validate the METTL3/NEU1 axis identified above, we performed in vitro assays. We found that sh-METTL3 reversed the upregulation of NEU1 and fibrosis markers in Ang II-induced atrial fibroblasts, while oe-NEU1 counteracted the effects of sh-METTL3 (Figure 5A–B). Furthermore, Ang II-induced proliferation and migration of atrial fibroblasts were inhibited by sh-METTL3, and oe-NEU1 reversed this inhibition (Figure 5C–D). These results demonstrate that METTL3 promotes Ang II-induced activation of atrial fibroblasts through NEU1.

## Discussion

Atrial fibrosis represents a fundamental pathological alteration in AF. Specifically, it disrupts normal electrical conduction, promotes the formation of reentrant circuits, and ultimately drives the progression of AF susceptibility. Although the importance of atrial fibrosis in AF has been widely recognized, the molecular mechanisms driving atrial fibrosis remain incompletely understood. In this study, we investigated the role of NEU1 in Ang II-induced AF susceptibility and the epigenetic mechanisms that regulate NEU1. Our findings demonstrate that NEU1 is significantly upregulated in atrial tissues of Ang II-treated mice (a model of AF susceptibility), promoting atrial fibrosis through enhanced fibroblast activation and ultimately increasing AF susceptibility.

NEU1 is expressed in the heart and plays an important role in cardiovascular diseases [21]. In this study, atrial tissues from Ang II-challenged mice exhibited elevated NEU1 levels, suggesting its potential involvement in AF susceptibility. Subsequent experiments in our study demonstrated that sh-NEU1 reversed Ang II-induced AF, indicating that NEU1 May play a promoting role in AF. Additionally, NEU1 is also implicated in fibrosis-related diseases, including renal fibrosis and diabetic myocardial fibrosis [8,10]. Importantly, our focus on NEU1 among the neuraminidase family (NEU1-4) is well justified. While NEU1 has been consistently implicated in fibrosis across multiple tissues and cardiovascular remodeling, other isoforms demonstrate distinct, non-overlapping pathophysiological roles: NEU2 is primarily associated with skeletal muscle metabolism [22]; NEU3 is mainly linked to cancer progression and lipid metabolism [23]; and NEU4 has been studied in the context of cancer, lysosomal storage disease, and cellular differentiation [24]. Critically, none of these other isoforms (NEU2-4) have established functional links to cardiac fibrosis or AF pathogenesis. Furthermore, our bioinformatic screening specifically identified high-confidence m6A modification sites on NEU1 mRNA, highlighting its unique potential as a novel target of epitranscriptomic regulation in atrial fibrosis – a mechanism not yet reported for other NEU isoforms in this context. Fibrotic atrial tissue remodeling drives both architectural and conductive abnormalities, creating the substrate for AF susceptibility. Therefore, we explored the role of NEU1 in atrial fibrosis. Animal experiments revealed that NEU1 knockdown reduced fibrosis in the atrial tissue of AF mice and inhibited Ang II-induced proliferation and migration of atrial fibroblasts. Atrial fibroblasts, often referred to as ‘sentinel cells,’ can detect chemical, mechanical, and electrical signals in the heart [25]. When the heart is injured, atrial fibroblasts migrate to the damaged area, secreting collagen and increasing extracellular matrix (ECM) deposition, leading to the expansion of the cardiac interstitium [25]. Under pathological conditions, atrial fibroblasts can further differentiate into myofibroblasts, promoting fibrosis [26,27]. Studies have reported that the largest structural component of the ECM is complex elastic fibers, and the generation of elastic fibers involves the sialidase activity of the NEU1 subunit [28]. Furthermore, the sialidase activity of NEU1 has been shown to promote the differentiation of fibroblasts into myofibroblasts [29]. Collectively, these observations validate NEU1 as a mediator of pro-fibrotic signaling in atrial fibroblasts, which contributes to AF susceptibility.

Regarding the downstream pathways mediating NEU1’s pro-fibrotic effects, based on existing literature, several interconnected pathways are likely involved. The TGF-β signaling pathway is a central candidate, as NEU1 can enhance TGFβRI activity via desialylation, activating Smad2/3 signaling [8]. Additionally, the AMPKα/SIRT3 oxidative stress pathway could form a pro-fibrotic positive feedback loop [10]. Future studies employing sialidase activity assays, pharmacological NEU1 inhibition (e.g., with Zanamivir/DANA), and detailed pathway interrogation are warranted to establish the core mediating mechanism.

M6A modification has been reported to be associated with AF, and several m6A regulatory genes play significant roles in AF susceptibility [30]. Through bioinformatics analysis, we identified highly credible m6A modification sites on NEU1, and subsequent molecular experiments confirmed that METTL3 mediates m6A modification of NEU1. The involvement of METTL3 in myocardial fibrogenesis has been well-documented in previous research [25]. Notably, the upregulation of METTL3 represents a common molecular response in profibrotic environments. Consistent with this, multiple experimental models – including TGF-β1-stimulated fibroblasts in vitro and fibrotic cardiac tissue in vivo – have demonstrated significantly elevated METTL3 expression [30]. Similarly, Ang II infusion, a well-established stimulus for cardiac fibrosis, also robustly induces METTL3 upregulation in atrial tissue [31], supporting the mechanistic relevance of METTL3 in Ang II-mediated fibrotic signaling. Silencing METTL3 inhibits cardiac fibroblast activation and fibrosis [30]. Studies by Tud et al. also demonstrated that METTL3 levels correlate with the degree of cardiac fibrosis in AF patients and that METTL3 induces cardiac fibrosis by enhancing the methylation of LncRNA GAS5 [30]. Additionally, overexpression of METTL3 in atrial fibroblasts significantly increases Collagen I and α-SMA expression, indicating the differentiation of atrial fibroblasts into myofibroblasts [25]. In agreement with established literature, our data demonstrate that METTL3 enhances the mRNA stability of NEU1 through m6A modification, and rescue experiments demonstrated that METTL3 promotes Ang II-induced atrial fibroblast activation by regulating NEU1 expression.

Despite revealing the pivotal contribution of NEU1 in Ang II-induced AF susceptibility and the molecular mechanism by which METTL3-mediated m6A modification regulates atrial fibrosis, there are some limitations. First, the findings are derived solely from an Ang II-infused mouse model and mouse atrial fibroblasts, and their relevance to human AF, especially non-hypertensive forms, requires future validation with clinical samples. Second, we lack direct morphological evidence (e.g., double immunofluorescence co-staining) to definitively localize NEU1 upregulation to atrial fibroblasts, although our functional data using fibroblast-specific NEU1 knockdown strongly support its primary role in this cell type. Third, while we demonstrated METTL3-mediated m6A modification of NEU1, the exact modification sites and functional impact remain to be mapped and validated. Fourth, our study focused on NEU1’s role in promoting atrial fibrosis as the arrhythmogenic substrate. We did not investigate its potential direct effects on cardiomyocyte electrophysiology or perform detailed electrophysiological mapping. Fifth, the use of only male mice limits the generalizability of our findings, given known sex differences in AF pathophysiology. Finally, although we employed a fibroblast-targeted knockdown approach, contributions from systemic Ang II effects (e.g., hemodynamic changes) to the observed phenotype cannot be entirely ruled out.

In summary, this study provides the inaugural evidence for the essential involvement of NEU1 in Ang II-induced AF susceptibility and elucidates the molecular mechanism by which METTL3 regulates NEU1 expression through m6A modification to promote atrial fibrosis. Our findings demonstrate that NEU1 is highly expressed in Ang II-treated mice and exacerbates atrial fibrosis by promoting the proliferation and migration of atrial fibroblasts, thereby increasing AF susceptibility. Furthermore, METTL3 enhances the mRNA stability of NEU1 through m6A modification, further confirming the important role of m6A modification in AF susceptibility. Beyond elucidating AF disease mechanisms, this work reveals potential biomarkers and intervention targets. Future studies should further explore the clinical significance of NEU1 in human AF and develop therapeutic strategies targeting NEU1 and its related signaling pathways, offering more effective treatment options for AF patients.

## Supplementary Material

supplementary table-1.docx

## Data Availability

The datasets used or analyzed during the current study are available from the corresponding author on reasonable request.

## List of Abbreviation

NEU1: Neuraminidase 1
NEU: Neuraminidase
AF: Atrial fibrillation
Ang II: Angiotensin II
METTL3: Methyltransferase-like 3
M6A: N6-methyladenosine
qPCR: Quantitative Reverse Transcription Polymerase Chain Reaction
ECL: Enhanced chemiluminescence
IF: Immunofluorescence
EdU: 5-Ethynyl-2′-deoxyuridine
RIP: RNA Immunoprecipitation
Postn: Periostin
