# Supplementary material for: N6-methyladenosine modification of NEU1 mediated by METTL3 exacerbates angiotensin II-induced atrial fibrillation
Source: Cell Adh Migr. 2026 Mar 31;20(1):2650272. doi: 10.1080/19336918.2026.2650272 (PMC13048583; doi:10.1080/19336918.2026.2650272)
Supplement: supplementary table-1.docx [file KCAM_A_2650272_SM7172.docx]

**Supplementary table**

**Table S1. Statistical results for Fig1A**

| Group | sample size |  | p-value | statistical test method |
| --- | --- | --- | --- | --- |
| Control | 3 | AngII vs. Control | <0.001 | Unpaired t test |
| AngII | 3 |  |  |  |

**Table S2. Statistical results for Fig1B**

| Group | sample size |  | p-value | statistical test method |
| --- | --- | --- | --- | --- |
| Control | 3 | AngII vs. Control | <0.001 | Unpaired t test |
| AngII | 3 |  |  |  |

**Table S3. Statistical results for Fig1D**

| Group | sample size |  | p-value | statistical test method |
| --- | --- | --- | --- | --- |
| Control | 8 | AngII vs. Control | <0.001 | Unpaired t test |
| AngII | 8 |  |  |  |

**Table S4. Statistical results for Fig2C**

| Group | sample size |  | p-value | statistical test method |
| --- | --- | --- | --- | --- |
| Control | 8 | AngII+sh-NC vs. Control | 0.0012 | Ordinary one-way ANOVA |
| AngII+sh-NC | 8 | AngII+sh-NEU1 vs. AngII+sh-NC | 0.0494 |  |
| AngII+sh-NEU1 | 8 |  |  |  |

**Table S5. Statistical results for Fig2D**

| Group | sample size |  | p-value | statistical test method |
| --- | --- | --- | --- | --- |
| Control | 8 | AngII+sh-NC vs. Control | <0.001 | Ordinary one-way ANOVA |
| AngII+sh-NC | 8 | AngII+sh-NEU1 vs. AngII+sh-NC | 0.004 |  |
| AngII+sh-NEU1 | 8 |  |  |  |

**Table S6. Statistical results for Fig2F**

| Group | sample size |  | p-value | statistical test method |
| --- | --- | --- | --- | --- |
| Control | 8 | AngII+sh-NC vs. Control | <0.001 | Ordinary one-way ANOVA |
| AngII+sh-NC | 8 | AngII+sh-NEU1 vs. AngII+sh-NC | <0.001 |  |
| AngII+sh-NEU1 | 8 |  |  |  |

**Table S7. Statistical results for Fig2G**

| Group | sample size |  | p-value | statistical test method |
| --- | --- | --- | --- | --- |
| Control | 8 | AngII+sh-NC vs. Control | <0.001 | Ordinary one-way ANOVA |
| AngII+sh-NC | 8 | AngII+sh-NEU1 vs. AngII+sh-NC | 0.003 |  |
| AngII+sh-NEU1 | 8 |  |  |  |

**Table S8. Statistical results for Fig2H**

**Collagen 1**

| Group | sample size |  | p-value | statistical test method |
| --- | --- | --- | --- | --- |
| Control | 3 | AngII+sh-NC vs. Control | 0.003 | Ordinary one-way ANOVA |
| Ang II+sh-NC | 3 | AngII+sh-NEU1 vs. AngII+sh-NC | 0.006 |  |
| Ang II+sh-NEU1 | 3 |  |  |  |

**α-SMA**

| Group | sample size |  | p-value | statistical test method |
| --- | --- | --- | --- | --- |
| Control | 3 | AngII+sh-NC vs. Control | 0.001 | Ordinary one-way ANOVA |
| Ang II+sh-NC | 3 | AngII+sh-NEU1 vs. AngII+sh-NC | 0.02 |  |
| Ang II+sh-NEU1 | 3 |  |  |  |

| **NEU1** |
| --- |

| Group | sample size |  | p-value | statistical test method |
| --- | --- | --- | --- | --- |
| Control | 3 | AngII+sh-NC vs. Control | <0.001 | Ordinary one-way ANOVA |
| Ang II+sh-NC | 3 | AngII+sh-NEU1 vs. AngII+sh-NC | 0.004 |  |
| Ang II+sh-NEU1 | 3 |  |  |  |

**Table S9. Statistical results for Fig3A**

**Collagen 1**

| Group | sample size |  | p-value | statistical test method |
| --- | --- | --- | --- | --- |
| Control | 3 | AngII+sh-NC vs. Control | <0.001 | Ordinary one-way ANOVA |
| Ang II+sh-NC | 3 | AngII+sh-NEU1 vs. AngII+sh-NC | <0.001 |  |
| Ang II+sh-NEU1 | 3 |  |  |  |

**α-SMA**

| Group | sample size |  | p-value | statistical test method |
| --- | --- | --- | --- | --- |
| Control | 3 | AngII+sh-NC vs. Control | 0.001 | Ordinary one-way ANOVA |
| Ang II+sh-NC | 3 | AngII+sh-NEU1 vs. AngII+sh-NC | 0.005 |  |
| Ang II+sh-NEU1 | 3 |  |  |  |

| **NEU1** |
| --- |

| Group | sample size |  | p-value | statistical test method |
| --- | --- | --- | --- | --- |
| Control | 3 | AngII+sh-NC vs. Control | <0.001 | Ordinary one-way ANOVA |
| Ang II+sh-NC | 3 | AngII+sh-NEU1 vs. AngII+sh-NC | 0.001 |  |
| Ang II+sh-NEU1 | 3 |  |  |  |

**Table S10. Statistical results for Fig3B**

**Collagen 1**

| Group | sample size |  | p-value | statistical test method |
| --- | --- | --- | --- | --- |
| Control | 3 | AngII+sh-NC vs. Control | 0.0016 | Ordinary one-way ANOVA |
| Ang II+sh-NC | 3 | AngII+sh-NEU1 vs. AngII+sh-NC | 0.0224 |  |
| Ang II+sh-NEU1 | 3 |  |  |  |

**α-SMA**

| Group | sample size |  | p-value | statistical test method |
| --- | --- | --- | --- | --- |
| Control | 3 | AngII+sh-NC vs. Control | 0.0016 | Ordinary one-way ANOVA |
| Ang II+sh-NC | 3 | AngII+sh-NEU1 vs. AngII+sh-NC | 0.022 |  |
| Ang II+sh-NEU1 | 3 |  |  |  |

| **NEU1** |
| --- |

| Group | sample size |  | p-value | statistical test method |
| --- | --- | --- | --- | --- |
| Control | 3 | AngII+sh-NC vs. Control | 0.0011 | Ordinary one-way ANOVA |
| Ang II+sh-NC | 3 | AngII+sh-NEU1 vs. AngII+sh-NC | 0.0188 |  |
| Ang II+sh-NEU1 | 3 |  |  |  |

**Table S11. Statistical results for Fig3C**

| Group | sample size |  | p-value | statistical test method |
| --- | --- | --- | --- | --- |
| Control | 3 | AngII+sh-NC vs. Control | 0.0013 | Ordinary one-way ANOVA |
| Ang II+sh-NC | 3 | AngII+sh-NEU1 vs. AngII+sh-NC | 0.0017 |  |
| Ang II+sh-NEU1 | 3 |  |  |  |

**Table S12. Statistical results for Fig3D**

| Group | sample size |  | p-value | statistical test method |
| --- | --- | --- | --- | --- |
| Control | 3 | AngII+sh-NC vs. Control | 0.001 | Ordinary one-way ANOVA |
| Ang II+sh-NC | 3 | AngII+sh-NEU1 vs. AngII+sh-NC | 0.001 |  |
| Ang II+sh-NEU1 | 3 |  |  |  |

**Table S13. Statistical results for Fig4A**

**atrial fibroblasts**

| Group | sample size |  | p-value | statistical test method |
| --- | --- | --- | --- | --- |
| Control | 3 | AngII vs. Control | 0.001 | Unpaired t test |
| Ang II | 3 |  |  |  |

**atrial tissues**

| Group | sample size |  | p-value | statistical test method |
| --- | --- | --- | --- | --- |
| Control | 3 | AngII vs. Control | <0.001 | Unpaired t test |
| Ang II | 3 |  |  |  |

**Table S14. Statistical results for Fig4B**

**atrial fibroblasts**

| Group | sample size |  | p-value | statistical test method |
| --- | --- | --- | --- | --- |
| Control | 3 | AngII vs. Control | 0.003 | Unpaired t test |
| Ang II | 3 |  |  |  |

**atrial tissues**

| Group | sample size |  | p-value | statistical test method |
| --- | --- | --- | --- | --- |
| Control | 3 | AngII vs. Control | 0.002 | Unpaired t test |
| Ang II | 3 |  |  |  |

**Table S15. Statistical results for Fig4C**

| Group | sample size |  | p-value | statistical test method |
| --- | --- | --- | --- | --- |
| IgG | 3 | METTL3 vs. lgG | 0.001 | Unpaired t test |
| METTL3 | 3 |  |  |  |

**Table S16. Statistical results for Fig4D**

| Group | sample size |  | p-value | statistical test method |
| --- | --- | --- | --- | --- |
| Control | 3 | Ang II+sh-NC vs. Control | <0.001 | Ordinary one-way ANOVA |
| Ang II+sh-NC | 3 | Ang II+sh-METTL3 vs. Ang II+sh-NC | <0.001 |  |
| Ang II+sh-METTL3 | 3 |  |  |  |

**Table S17. Statistical results for Fig4E**

| Group | sample size |  | p-value | statistical test method |
| --- | --- | --- | --- | --- |
| Control | 3 | Ang II+sh-NC vs. Control | 0.007 | Ordinary one-way ANOVA |
| Ang II+sh-NC | 3 | Ang II+sh-METTL3 vs. Ang II+sh-NC | 0.04 |  |
| Ang II+sh-METTL3 | 3 |  |  |  |

**Table S18. Statistical results for Fig4F**

| Group | sample size |  | p-value | statistical test method |
| --- | --- | --- | --- | --- |
| sh-NC | 3 | sh-METTL3 vs. sh-NC | 0.0061 | Unpaired t test |
| sh-METTL3 | 3 |  |  |  |

**Table S19. Statistical results for Fig5A**

**Collagen 1**

| Group | sample size |  | p-value | statistical  test method |
| --- | --- | --- | --- | --- |
| Control | 3 | Ang II+sh-NC vs. Control | <0.001 | Ordinary one-way ANOVA |
| Ang II+sh-NC | 3 | Ang II+sh-METTL3 vs. Ang II+sh-NC | 0.006 |  |
| Ang II+sh-METTL3 | 3 | Ang II+sh-METTL3+oe-NEU1 vs.  Ang II+sh-METTL3 | 0.01 |  |
| Ang II+sh-METTL3  +oe-NEU1 | 3 |  |  |  |

**α-SMA**

| Group | sample size |  | p-value | statistical  test method |
| --- | --- | --- | --- | --- |
| Control | 3 | Ang II+sh-NC vs. Control | 0.001 | Ordinary one-way ANOVA |
| Ang II+sh-NC | 3 | Ang II+sh-METTL3 vs. Ang II+sh-NC | 0.02 |  |
| Ang II+sh-METTL3 | 3 | Ang II+sh-METTL3+oe-NEU1 vs.  Ang II+sh-METTL3 | 0.001 |  |
| Ang II+sh-METTL3  +oe-NEU1 | 3 |  |  |  |

**NEU1**

| Group | sample size |  | p-value | statistical  test method |
| --- | --- | --- | --- | --- |
| Control | 3 | Ang II+sh-NC vs. Control | <0.001 | Ordinary one-way ANOVA |
| Ang II+sh-NC | 3 | Ang II+sh-METTL3 vs. Ang II+sh-NC | <0.001 |  |
| Ang II+sh-METTL3 | 3 | Ang II+sh-METTL3+oe-NEU1 vs.  Ang II+sh-METTL3 | 0.001 |  |
| Ang II+sh-METTL3  +oe-NEU1 | 3 |  |  |  |

**Table S20. Statistical results for Fig5B**

**Collagen 1**

| Group | sample size |  | p-value | statistical  test method |
| --- | --- | --- | --- | --- |
| Control | 3 | Ang II+sh-NC vs. Control | <0.001 | Ordinary one-way ANOVA |
| Ang II+sh-NC | 3 | Ang II+sh-METTL3 vs. Ang II+sh-NC | <0.001 |  |
| Ang II+sh-METTL3 | 3 | Ang II+sh-METTL3+oe-NEU1 vs.  Ang II+sh-METTL3 | 0.01 |  |
| Ang II+sh-METTL3  +oe-NEU1 | 3 |  |  |  |

**α-SMA**

| Group | sample size |  | p-value | statistical  test method |
| --- | --- | --- | --- | --- |
| Control | 3 | Ang II+sh-NC vs. Control | <0.001 | Ordinary one-way ANOVA |
| Ang II+sh-NC | 3 | Ang II+sh-METTL3 vs. Ang II+sh-NC | 0.005 |  |
| Ang II+sh-METTL3 | 3 | Ang II+sh-METTL3+oe-NEU1 vs.  Ang II+sh-METTL3 | 0.03 |  |
| Ang II+sh-METTL3  +oe-NEU1 | 3 |  |  |  |

**NEU1**

| Group | sample size |  | p-value | statistical  test method |
| --- | --- | --- | --- | --- |
| Control | 3 | Ang II+sh-NC vs. Control | <0.001 | Ordinary one-way ANOVA |
| Ang II+sh-NC | 3 | Ang II+sh-METTL3 vs. Ang II+sh-NC | <0.001 |  |
| Ang II+sh-METTL3 | 3 | Ang II+sh-METTL3+oe-NEU1 vs.  Ang II+sh-METTL3 | 0.04 |  |
| Ang II+sh-METTL3  +oe-NEU1 | 3 |  |  |  |

**Table S21. Statistical results for Fig5C**

| Group | sample size |  | p-value | statistical  test method |
| --- | --- | --- | --- | --- |
| Control | 3 | Ang II+sh-NC vs. Control | <0.001 | Ordinary one-way ANOVA |
| Ang II+sh-NC | 3 | Ang II+sh-METTL3 vs. Ang II+sh-NC | 0.001 |  |
| Ang II+sh-METTL3 | 3 | Ang II+sh-METTL3+oe-NEU1 vs.  Ang II+sh-METTL3 | 0.008 |  |
| Ang II+sh-METTL3  +oe-NEU1 | 3 |  |  |  |

**Table S22. Statistical results for Fig5D**

| Group | sample size |  | p-value | statistical  test method |
| --- | --- | --- | --- | --- |
| Control | 3 | Ang II+sh-NC vs. Control | <0.001 | Ordinary one-way ANOVA |
| Ang II+sh-NC | 3 | Ang II+sh-METTL3 vs. Ang II+sh-NC | 0.001 |  |
| Ang II+sh-METTL3 | 3 | Ang II+sh-METTL3+oe-NEU1 vs.  Ang II+sh-METTL3 | 0.002 |  |
| Ang II+sh-METTL3  +oe-NEU1 | 3 |  |  |  |
